# Does a physician-led Helicopter Emergency Medical Service (HEMS) favour its base hospital and interfere with agreed local trauma network ‘Trauma Decision Trees’?

**DOI:** 10.1186/1757-7241-20-S1-P1

**Published:** 2012-03-22

**Authors:** O Maunsell, R Wen, A Weaver, D Lockey

**Affiliations:** 1 University of Newcastle Upon Tyne, UK; 2 University of Melbourne, Australia; 3 The Royal London Hospital, UK

## Background

London’s ‘Major Trauma Network’ was established in April 2010, and comprises four ‘Major Trauma Centres’ (MTCs) – each offering all trauma-related specialties under one roof.

Healthcare for London produced an algorithmic ‘Major Trauma Decision Tree’ to help emergency healthcare professionals decide where to convey patients, in order to offer the most suitable care for their needs.

The tree states that trauma patients who meet certain clinical criteria are to be conveyed to the nearest MTC.

## Objective

To assess the adherence to the Major Trauma Decision Tree by London HEMS when conveying patients to the Royal London Hospital (RLH) by road over a 6-month period.

## Design

Retrospective Audit.

## Methods

489 patient records were examined; all of whom had been conveyed to the RLH by London HEMS over a 6-month period (1/7/10 – 31/12/10).

Distances were calculated from each job to all of the MTCs. These were converted into road times using an online journey planner, and then into blue-light times by applying a conversion factor.

Cases where the destination choice was not the nearest MTC (ie a ‘breach’) were placed into one of five justification categories.

## Results

London HEMS was, in 97% of its land conveyances of major trauma patients, compliant with the decision tree. The 17 ‘breaches’ were all justifiable; the most common justification being a negligible difference in time to MTCs (less than 5 minutes).

## Conclusion

There is no evidence of ‘favouritism’ to any of the MTCs by London HEMS, and compliance to the decision tree is extremely high.

